# Diabetes Mellitus and Infertility: Different Pathophysiological Effects in Type 1 and Type 2 on Sperm Function

**DOI:** 10.3389/fendo.2018.00268

**Published:** 2018-05-25

**Authors:** Rosita A. Condorelli, Sandro La Vignera, Laura M. Mongioì, Angela Alamo, Aldo E. Calogero

**Affiliations:** Department of Clinical and Experimental Medicine, University of Catania, Catania, Italy

**Keywords:** type 1 diabetes mellitus, type 2 diabetes mellitus, infertility, conventional sperm parameters, biofunctional sperm parameters, sperm function

## Abstract

Although the prevalence of sub-infertility in diabetic patients in childbearing age is known, the mechanisms by which diabetes mellitus (DM) causes male infertility are not completely explained. This detrimental effect is achieved with a variety of mechanisms that include pre-testicular, testicular, and post-testicular pathogenetic moments and can be different in type 1 diabetes mellitus (DM1) and type 2 diabetes mellitus (DM2) patients because of type of diabetes, duration of disease, and glycemic metabolic compensation. Aim of this study was to evaluate whether diabetic disease can be considered a risk factor for infertility considering the etiopathogenetic differences between DM1 and DM2 on sperm function. We enrolled 38 DM1 patients and 55 DM2 patients with idiopathic infertility history >12 months, and 100 healthy fertile subjects. The following outcomes were evaluated in optical microscopy and flow cytometry: sperm function (by conventional and biofunctional sperm parameters) and signs of urogenital infection/inflammation (by sperm leukocyte concentrations and indices of oxidative stress). Moreover, an andrological evaluation (by didymo-epididymal ultrasound evaluation, serum total testosterone, LH, and FSH measurements) was performed in DM1 and DM2 patients compared to controls. Diabetic patients showed a higher risk of becoming infertile and the pathophysiological mechanisms of damage were different in DM1 and DM2. Conventional sperm parameters of diabetic patients are worse than controls (*p* < 0.05). The DM2 caused an inflammatory condition with increased oxidative stress resulting in decreased sperm vitality and increased sperm DNA fragmentation. DM1 altered epididymal voiding causing low ejaculate volume and mitochondrial damage resulting in decreased sperm motility. These findings and evidences support the contention that DM could be regarded as cause of male infertility suggesting that the prevention of diabetic disease in DM2 and the follow-up of seminal parameters in DM1 could prevent fertility decline in these categories of patients.

## Introduction

An increased number of male patients with diabetes mellitus (DM) have been reported in childbearing age and the DM prevalence is closely associated with the decline of fertility (1, 2). In addition, both type 1 diabetes mellitus (DM1) and type 2 diabetes mellitus (DM2) involve an alarming number of children and adolescents (3). The rising proportion of people aged 40 years or younger at diagnosis with DM2 is particularly interesting and worrying because leads to longer lifetime exposure to hyperglycemia and long-term complications (4). So, the early course of DM2 in young people could be more disruptive leading to poor quality of life as well as in DM1 patients: evidences suggest that loss of β-cell function is accelerated in young-onset DM2 and prevalence estimates show a 31% increase already in DM2 among people aged 10–19 years (4). Therefore, these epidemiological data suggest that the diabetic disease arrives even before the desire to have a child.

A review of experimental and clinical studies shows that DM is associated with worse conventional sperm parameters, without to reach particularly low values (5, 6). DM1 is able to influence the expression of genes involved in sperm DNA repair, resulting in a high rate of nuclear DNA fragmentation (7), mitochondrial DNA deletions (5, 6, 8, 9) with mitochondrial respiratory chain alteration and subsequent decreased sperm motility (10).

It has been shown that sperm plasma membrane and acrosome are affected by serum insulin levels (11). Therefore, during insulin resistance or insulin deficiency, spermatogenesis is altered and biopsies of diabetic patients reveal testicular alterations (12).

Diabetes mellitus may potentially cause male infertility with a pre-testicular, testicular, and post-testicular mechanism. In the first, DM patients develop hypogonadism through central (hyperleptinemia or alterations of hypothalamic GnRH pulsatile release in overweight or obese patients) (13) and peripheral (alteration of Leydig cell function) (14) mechanisms resulting in lower serum gonadotropin and testosterone levels.

At testicular level, DM seems to cause: (a) increased oxidative stress with increased reactive oxygen species production in seminal fluid (15) and lipoperoxidation (LP); (b) sperm DNA fragmentation (8); (c) sperm mitochondrial bioenergy alteration (8); and (d) enzymatic glycation end products (16).

Moreover, possible mechanisms post-testicular can occur because DM may cause sperm damage and/or prevent seminal fluid release by (a) male accessory gland infection/inflammation (MAGI) (17, 18) where the DM-MAGI association amplifies the inflammatory response in semen (19) altering conventional sperm parameters and favoring a greater extension of the inflammatory process and its chronicization (20, 21) and (b) erectile and/or ejaculatory dysfunction, well-known complications of DM.

The aim of this study was to evaluate the different pathophysiological mechanisms of sperm damage in DM1 and DM2 patients in childbearing age, with idiopathic infertility, assessing conventional and biofunctional sperm parameters and oxidative stress indices, the presence of urogenital infection/inflammation, and an andrological evaluation in both groups of patients.

## Materials and Methods

### Patient Selection

The patients were divided into three groups: 38 patients with DM1, 55 with DM2 [according to the Standards of Medical Care in Diabetes (22)] with idiopathic infertility history >12 months [according to the International Glossary on Infertility and Fertility Care (23)], and a sample of 100 healthy fertile subjects as “controls.”

All diabetic patients were investigated according to glycemic metabolic compensation. DM1 patients were divided into three groups according to the duration of the disease (<5 years, between 5–10 years, and >10 years). DM2 patients were not subdivided according to duration of illness to avoid bias related due to age-related disease. This is because DM2 occurs temporally after DM1 and all patients enrolled were in childbearing age. DM2 patients reported a previous nonalcoholic steatohepatitis returned after weight loss.

All enrolled subjects did not smoke, drink alcohol, and did not use drugs. They did not have male accessory glands infection, systemic diseases, microorchidism (testicular volume < 12 ml), cryptorchidism, varicocele, and did not receive hormonal treatment in the last 12 months.

This study was approved by the Ethics Committee of University teaching Hospital of “Policlinico-Vittorio Emanuele,” University of Catania (Catania, Italy), trial registration number 104/2016/PO (Register of the Ethics Committee opinions). All methods were performed in accordance with the relevant guidelines and regulations. All participants were asked for and provided their informed consent.

### Experimental Design

Each enrolled subject underwent to a sperm analysis (conventional sperm parameters), a flow cytometric sperm analysis (biofunctional sperm parameters), the assessment of the presence of urogenital infection/inflammation, oxidative stress evaluation, and an andrological evaluation.

Assessment of the presence of urogenital infection/inflammation was performed by the concentration of peroxidase-positive leukocytes evaluated by optical microscopy (24) and whenever the leukocytes were above the normal range, the identification of leukocyte subpopulations in seminal fluid was observed by cytofluorimetric analysis to classify the urogenital infection/inflammation (bacterial, viral, acute, or chronic) (25).

Oxidative stress evaluation in the seminal fluid was executed by measuring the degree of mitochondrial superoxide and the degree of LP.

Andrological evaluation of these patients was performed by didymo-epididymal ultrasound evaluation before and after ejaculation for the evaluation of the degree of epididymal voiding and contractility and the serum total testosterone (TT), LH, and FSH determination to identify the possible presence of hypogonadism and/or erectile dysfunction and to exclude pre-existing testicular damages.

Seminal analysis of each sample was replicated as reported by the WHO 2010 guidelines.

### Sperm Analysis and Preparation

Semen samples were collected by masturbation into a sterile container after 2–7 days of sexual abstinence and were transported to the laboratory within 30 min after ejaculation. Each sample was evaluated for seminal volume, pH, viscosity, sperm count, progressive motility, morphology, and round cell concentration (spermatids and leukocytes) according to the WHO criteria (24).

### Flow Cytometric Analysis

Flow cytometric analysis was performed using flow cytometer EPICS XL (Coulter Electronics, IL, USA/Milan, Italy) equipped with an argon laser at 488 nm. We used the FL1 detectors for the green (525 nm), FL2 for the orange (575 nm), and FL3 for the red (620 nm) fluorescence; 100,000 events (low velocity) were measured for each sample and analyzed by the software Sistem II™, Version 3.0. The debris was gated out, by drawing a region on forward vs. side scatter dot plot enclosing the population of cells of interest. Computed compensation was made before performing all the analyses.

#### Evaluation of the Mitochondrial Membrane Potential (MMP)

The damage of MMP is an early event of the apoptosis and it is reversible. MMP can be evaluated using the lipophilic probe 5,5′,6,6′-tetrachloro-1,1′,3,3′tetraethyl-benzimidazolylcarbocyanine iodide (JC-1, DBA s.r.l., Milan, Italy). JC-1 is able to penetrate selectively in mitochondria and it exists in monomeric form, emitting at 527 nm; following excitation at 490 nm and in relation to the membrane potential, JC-1 is able to form aggregates emitting at 590 nm. The fluorescence changes reversibly from green to orange as soon as the mitochondrial membrane becomes more polarized. Therefore, it is possible to distinguish two cell populations: cells with damaged MMP where JC-1 remains in the cytoplasm in a monomeric form, giving a green fluorescence and cells with normal MMP with a double fluorescence where JC-1 (in addition to emitting green in the cytosol) is also in the mitochondrial membrane in form of aggregates emitting in an orange fluorescence. An aliquot containing 1 × 10^6^/ml of spermatozoa were incubated with JC-1 in the dark, for 10 min, at a temperature of 37°C. At the end of the incubation period, the cells were washed in phosphate buffered saline (PBS) and analyzed by the detectors FL1 and FL3.

#### Assessment of the Degree of Chromatin Compactness

The evaluation of chromatin integrity was performed after permeabilization of the cell membrane, to allow the access of the fluorophore within the nucleus. An aliquot of 1 × 10^6^ spermatozoa was incubated with LPR DNA-Prep Reagent containing 0.1% potassium cyanate, 0.1% NaN_3_, non-ionic detergents, saline, and stabilizers (Beckman Coulter, IL, USA/Milan, Italy), in the dark, at room temperature for 10 min and then further incubated with Stain DNA-Prep Reagent containing 50 µg/ml of propidium iodide (PI) (<0.5%), RNase A (4 KU/ml), <0.1% NaN3, saline, and stabilizers (Beckman Coulter, IL, USA) in the dark at room temperature. Flow cytometric analysis was performed after 30 min, using FL3 detector.

#### Evaluation of Sperm Apoptosis/Vitality

The externalization of phosphatidylserine (PS) on the outer cell surface is an early signal of apoptosis. The assessment of PS externalization was performed using annexin V, protein that binds selectively to PS in presence of calcium ions, FITC-labeled. During apoptosis, the cells exhibiting the PS even before the loss of semipermeability. Therefore, marking simultaneously the cells with annexin V and PI, we could distinguish: alive (with intact cytoplasmic membrane), apoptotic, or necrotic cells. Staining with annexin V and PI was obtained using a commercially available kit (Annexin V-FITC Apoptosis, Beckman Coulter, IL, USA/Milan, Italy).

An aliquot containing 0.5 × 10^6^/ml was suspended in 0.5 ml of buffer containing 10 µl of annexin V-FITC and 20 µl of PI and incubated for 10 min in the dark. After incubation, the sample was analyzed immediately by the detectors FL1 (FITC) and FL3 (PI). The different pattern of staining allowed to identify the different cell populations: FITC negative and PI negative indicate viable cells, FITC positive and PI negative indicate cells in early apoptosis with cytoplasmic membrane still intact, and FITC positive and PI positive indicate cells in late apoptosis.

#### Assessment of DNA Fragmentation

The evaluation of DNA fragmentation was performed by the TUNEL method. This uses the Terminal deoxynucleotidyl Transferase (TdT), an enzyme that polymerizes, at the level of DNA breaks, modified nucleotides conjugated to a fluorochrome. The TUNEL assay was performed by using a commercially available kit (DBA s.r.l., Milan, Italy). To obtain a negative control, TdT was omitted from the reaction mixture; the positive control was obtained pretreating spermatozoa (about 0.5 × 10^6^) with 1 mg/ml of deoxyribonuclease I, not containing RNAse, at 37°C for 60 min prior to staining. The debris was gated out as described above. The reading by threshold-setting method was performed by flow cytometry using the FL1 detector.

### Oxidative Stress Evaluation

#### Evaluation of Sperm Lipid Peroxidation

LP evaluation by flow cytometry was performed using the probe, BODIPY (581/591) C11 (Invitrogen, Thermo Fisher Scientific, Eugene, OR, USA) which after being incorporated into cell membranes, responds to the attack of free oxygen radicals changing its spectrum of emission from red to green. This displacement of the emission is shown by the flow cytometer which provides an estimate of the degree of peroxidation.

LP was evaluated in two different sperm aliquots of the same patient: the first consisting of spermatozoa separated by swim-up and the second obtained by centrifugation of the seminal fluid (raw semen). About 2 × 10^6^ of spermatozoa were incubated with 5 mM of the probe for 30 min in a final volume of 1 ml. After washing with PBS, flow cytometric analysis was conducted using the FL1 and FL2 detectors.

#### Determination of Sperm Mitochondrial Superoxide Levels

Mitochondrial superoxide levels were detected by the MitoSOX red mitochondrial superoxide indicator (Invitrogen, Thermo Fisher Scientific, Eugene, OR, USA) (26). This probe, once penetrated into the mitochondria, is quickly oxidized by superoxide anion (not from other free radicals) and as a result of this process, the probe becomes highly fluorescent with signal detection. About 1 × 10^6^ of spermatozoa were incubated with 5 µM of the probe for 10 min in a final volume of 1 ml and at a temperature of 37°C. After washing with PBS, flow cytometric analysis was conducted using FL1 detectors.

#### Evaluation of Leukocyte Subpopulation

Leukocyte subpopulations were identified by flow cytometry using monoclonal antibodies (Beckman Coulter, IL, USA/Milan, Italy) against specific membrane antigens. Specifically, after liquefaction, three aliquots of 100 µl of seminal fluid underwent to three washing cycles with PBS. After elimination of the supernatant, the pellet was resuspended in 1 ml of PBS. Then:

- the first aliquot was incubated with Ab anti CD45 (to identify leukocyte population), Ab anti CD16 for neutrophils (CD45 and CD16 co-expression), and Ab anti CD14 for monocyte-macrophages (co-expression of CD45 and CD14);
- the second aliquot was incubated with Ab anti CD45, Ab anti CD3, and Ab anti CD4 (to identify T-helper lymphocytes);
- the third aliquot was incubated with Ab anti CD45, Ab anti CD3, and Ab anti CD8 (to identify T-suppressor lymphocytes).

Flow cytometric analysis was conducted using FL1, FL2, and FL3 detectors.

### Ultrasound Evaluation

All patients underwent didymo-epididymal ultrasound evaluation using a linear 7.5 MHz (Esaote MyLab25, Genova, Italy). Ultrasound evaluation of the testicular volume and epididymal diameters was performed, before and after ejaculation, by the same clinician (SLV). The ultrasound parameters evaluated were epididymal caput (CEAE) and tail (TEAE) after ejaculation, testicular volume (VT), cranial (CEBE), and caudal (TEBE) diameter before ejaculation. The operator repeated twice the measurement of these parameters are expressed as mean on the final report.

### Hormone Serum Determination

Blood sampling was performed at 8.00 a.m. Determination of LH, FSH, and TT serum levels was performed by electrochemiluminescence immunoassay with Cobas equipment.

### Statistical Analysis

The results are expressed as mean ± SEM throughout the study. The data were analyzed by one-way analysis of variance followed by the Duncan’s Multiple Range test. SPSS 22.0 for Windows was used for statistical analysis (SPSS Inc., Chicago, IL, USA). Statistical significance was accepted when the *p* value was lower than 0.05.

## Results

The anthropometric parameters of the patients enrolled in this study are shown in Table 1. DM patients showed no statistically significant differences in age, BMI, and waist circumference compared to controls (Table 1).

**Table 1:** Anthropometric parameters of patients with type 1 diabetes mellitus (DM1), type 2 diabetes mellitus (DM2), and the control (CTL) group.

	CTL (*n* = 100)	DM1 (*n* = 38)	DM2 (*n* = 55)
Age	28.0 ± 2.7	27.0 ± 1.9	29.0 ± 1.5
BMI (kg/m^2^)	26.2 ± 1.3	26.5 ± 0.9	25.9 ± 1.4
Waist circumference (cm)	95.0 ± 2.8	94.0 ± 2.2	97.0 ± 2.4

### Conventional and Biofunctional Sperm Parameters

**Figure 1:**
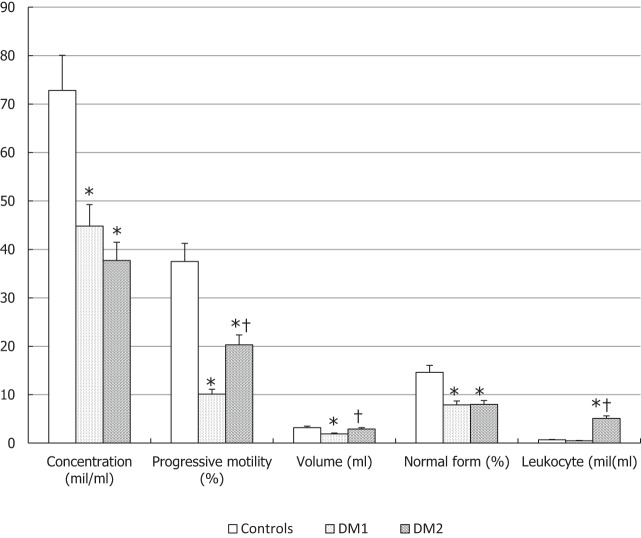
Conventional sperm parameters in patients with type 1 diabetes mellitus (DM1), type 2 diabetes mellitus (DM2), and the control (CTL) group. *p < 0.05 vs. CTL; ^†^*p* < 0.05 vs. DM1. The results are expressed as mean ± SEM.

Conventional sperm parameters were significantly different in the three groups of patients (Figure 1). Patients with DM1 or DM2 showed a statistically significant decrease in sperm concentration as compared to controls and DM2 patients had a slightly lower sperm concentration than DM1 patients. Progressive motility was lower in patients with DM1 and DM2 compared to controls (*p* < 0.05), and it was lower in DM1 than in DM2 patients (*p* < 0.05). Seminal fluid volume was significantly lower in patients with DM1 (*p* < 0.05), while it did not seem to undergo significant changes in DM2 patients compared to controls. Diabetic patients showed a lower percentage of spermatozoa with normal form compared to controls (*p* < 0.05). The concentration of peroxidase-positive leukocytes evaluated by optical microscopy was significantly higher in patients with DM2 than in the two groups (*p* < 0.05) (Figure 1).

**Figure 2:**
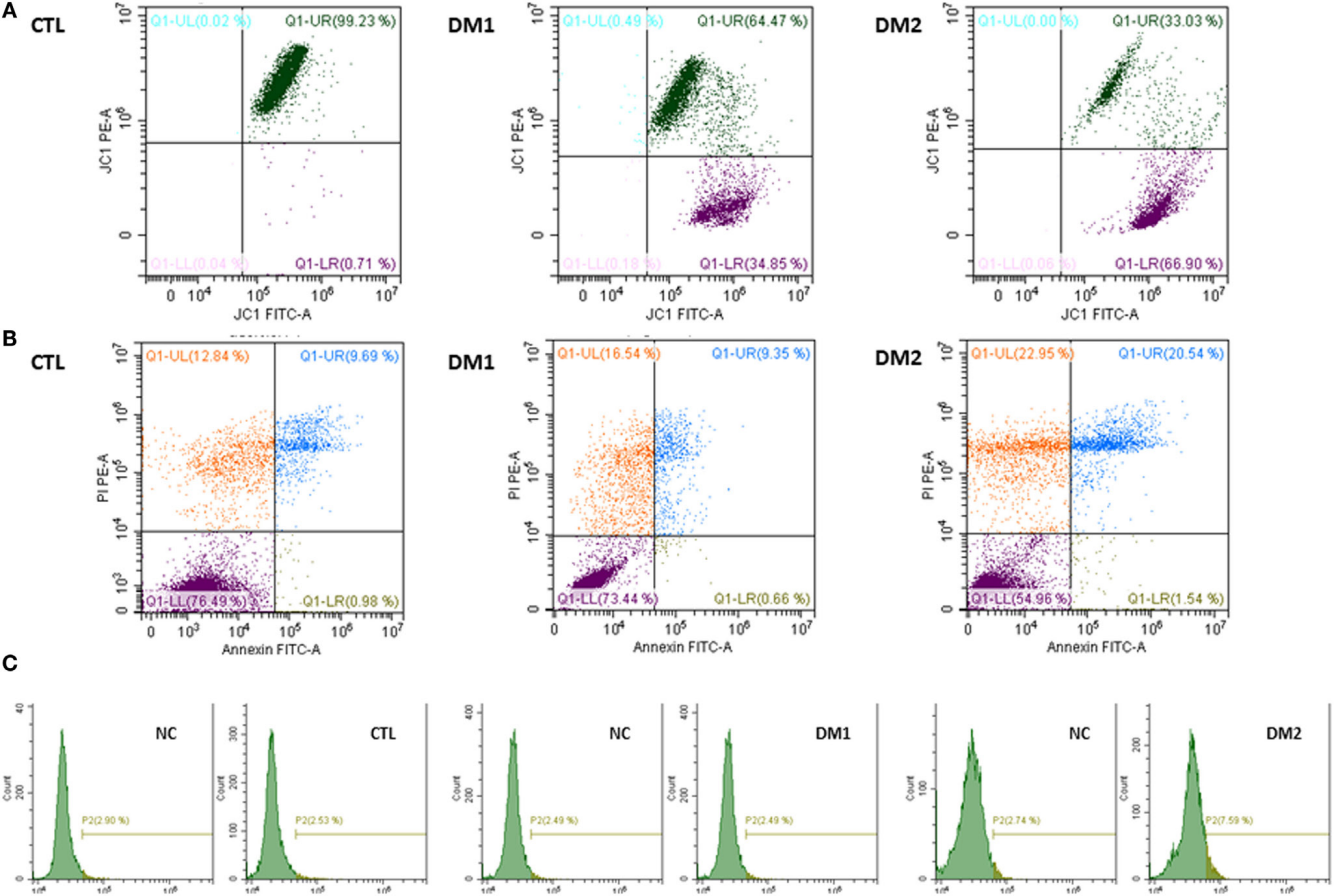
Dot plot related to flow cytometric evaluation of mitochondrial membrane potential in a control, in a patient with type 1 diabetes mellitus (DM1), and in a patient with type 2 diabetes mellitus (DM2) **(A)**. Dot plot related to flow cytometric evaluation of vitality/apoptosis in a control, in a DM1 patient and in a DM2 patient **(B)**. Dot plot related to DNA fragmentation, with negative control (NC), for threshold-setting method, in a control, in a patient with DM1, and in a patient with DM2 **(C)**.

In Figure 2, we show dot plots related to flow cytometric evaluation of MMP, vitality/apoptosis, and DNA fragmentation of a control, a DM1 patient and DM2 patient (Figure 2).

**Figure 3:**
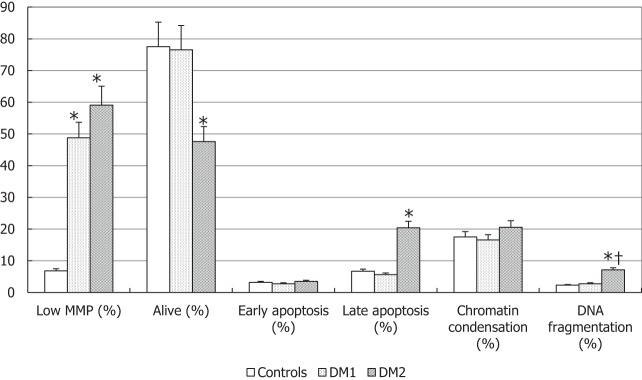
Biofunctional sperm parameters in patients with type 1 diabetes mellitus (DM1), type 2 diabetes mellitus (DM2), and control (CTL) group. *p < 0.05 vs. CTL; ^†^*p* < 0.05 vs. DM1. The results are expressed as mean ± SEM.

The evaluation of biofunctional sperm parameters (Figure 3) showed that the percentage of spermatozoa with low MMP was higher in both groups of diabetic patients (*p* < 0.05).

Only DM2 patients showed a significant decrease of sperm vitality with a concomitant increase of spermatozoa in late apoptosis compared to controls (*p* < 0.05), whereas no significant variation was observed for the other biofunctional parameters, such as early apoptosis and degree of chromatin compaction. Finally, patients with DM2 had a higher percentage of DNA fragmentation spermatozoa compared to DM1 patients and controls (*p* < 0.05) (Figure 3).

Type 1 diabetes mellitus patients showed a lower progressive motility when the duration of illness was longer than 10 years compared to the other two groups with less duration of illness (*p* < 0.05) (Table 2). The other conventional sperm parameters did not appear significantly different. Only spermatozoa with low MMP parameter were higher in DM1 patients after 5 years of disease (*p* < 0.05). The other biofunctional sperm parameters did not vary significantly (Table 2). HbA1c levels did not correlate with any of the examined parameters (data not shown).

**Table 2:** Conventional and biofunctional sperm parameters in patients with type 1 diabetes mellitus (DM1) according to the duration of the disease.

	DM1 < 5 years	DM1 5–10 years	DM1 > 10 years
Concentration (mil/ml)	42.8 ± 12.0	42.5 ± 11.9	42.0 ± 11.5
Progressive motility (%)	11.2 ± 3.3	10.5 ± 3.5	6.5 ± 3.4^†^
Normal form (%)	8.3 ± 0.8	8.2 ± 0.9	7.9 ± 1.5
Low mitochondrial membrane potential (%)	41.3 ± 10.2	52.5 ± 9.9*	54.1 ± 10.1*
Early apoptosis (%)	1.7 ± 0.1	1.9 ± 0.1	1.8 ± 0.1
Chromatin condensation (%)	15.5 ± 2.3	15.2 ± 2.1	14.7 ± 2.0
DNA fragmentation (%)	2.3 ± 0.5	3.1 ± 0.2	2.5 ± 0.3

^†^p < 0.05 vs. DM1 < 10 years; *p < 0.05 vs. DM1 < 5 years.

### Cytofluorimetric Parameters of Urogenital Inflammation

**Figure 4:**
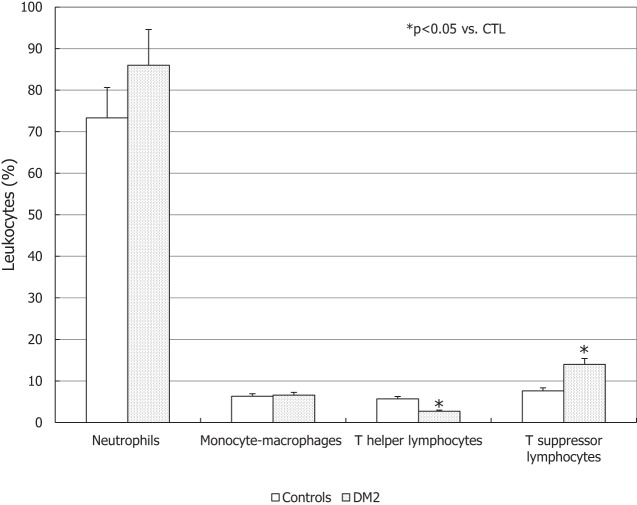
Leukocyte subpopulations in the seminal fluid of patients with type 2 diabetes mellitus (DM2) and controls (CTL). *p < 0.05 vs. CTL. Data are expressed as mean ± SEM.

The percentage of neutrophils and monocyte-macrophages was similar in the DM2 group compared to controls, whereas a significant decrease of the percentage of T-helper lymphocytes and an increase in T-suppressor lymphocytes was found in the seminal fluid of DM2 patients compared to controls (*p* < 0.05) (Figure 4).

### Oxidative Stress Evaluation

**Figure 5:**
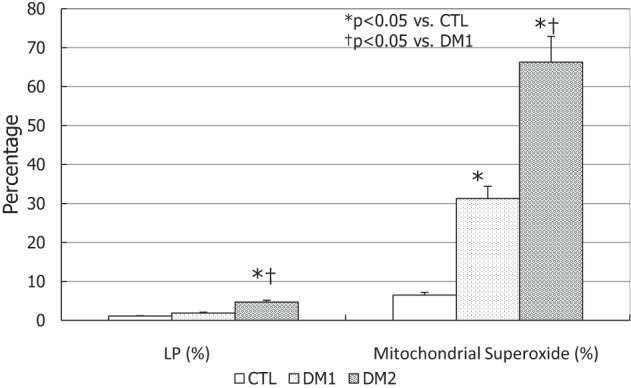
Lipid peroxidation (LP) and mitochondrial superoxide concentrations type 1 diabetes mellitus (DM1), type 2 diabetes mellitus (DM2), and controls (CTL) in seminal fluid. *p < 0.05 vs. CTL; ^†^*p* < 0.05 vs. DM1. The results are expressed as mean ± SEM.

The degree of LP was higher in DM2 patients compared to the other two groups (*p* < 0.05), whereas the mitochondrial superoxide concentrations were higher in DM patients than in controls and, in particular, in DM2 compared to DM1 (*p* < 0.05) (Figure 5).

### Ultrasound Parameters

CEAE and TEAE appeared to be greater when the duration of illness was longer than 10 years (*p* < 0.05), whereas no difference was found in the VT, CEBE, and TEBE (Table 3).

**Table 3:** Didymo-epididymal parameters in patients with type 1 diabetes mellitus (DM1) according to the duration of the disease.

	DM1 < 5 years	DM1 5–10 years	DM1 > 10 years
TV (ml)	21.0 ± 3.2	22.0 ± 2.1	20.0 ± 2.5
CEBE (mm)	12.2 ± 1.3	12.0 ± 1.1	12.5 ± 1.4
CEAE (mm)	9.0 ± 0.8	9.2 ± 0.8	12.0 ± 1.1*
TEBE (mm)	5.2 ± 0.5	6.3 ± 0.6	5.3 ± 0.7
TEAE (mm)	3.1 ± 0.4	3.3 ± 0.6	5.2 ± 0.8*

*TV, testicular volume; CEBE, cranial epididymal diameter before ejaculation; CEAE, caput epididymal diameter after ejaculation; TEBE, tail epididymal diameter before ejaculation; TEAE, tail epididymal diameter after ejaculation*.

*p < 0.05 vs. DM1 < 10 years.

### Hormone Serum Measurements

LH was higher in patients with DM2 than in the other groups (*p* < 0.05) and TT serum levels were lower in DM2 patients compared to other groups though not statistically significant (Table 4). FSH was not reveal significantly different in DM patients compared to controls (Table 4). When patients with DM1 were classified according to the duration of the disease, no statistically significant difference was observed among three groups (data not shown).

**Table 4:** Hormone concentrations of patients with type 1 diabetes mellitus (DM1), type 2 diabetes mellitus (DM2), and the control (CTL) group.

	CTL (*n* = 100)	DM1 (*n* = 38)	DM2 (*n* = 55)
Total testosterone (ng/ml)	5.1 ± 0.1	6.1 ± 1.1	4.1 ± 0.5
LH (UI/l)	2.4 ± 0.1	2.1 ± 0.1	5.6 ± 0.1*
FSH (UI/l)	2.9 ± 0.9	2.3 ± 0.3	2.5 ± 0.1

*p < 0.05 vs. other groups.

## Discussion

The results of this study showed that diabetic patients in childbearing age have alterations of conventional sperm parameters compared to non-diabetic controls. Of particular, the main alterations concern sperm concentration, progressive motility, and normal forms. Patients with DM1 showed a progressive motility that significantly deteriorated alongside the duration of the disease. Previous studies did not show significant variations of this parameter and no correlation with age, disease duration, or HbA1c levels (8, 27). By contrast, other studies confirm our data showing a decrease in sperm progressive motility in patients with DM1 and DM2 (28) and in 32 DM1 teenage patients (29).

Mitochondrial membrane potential is the main parameter that better reflects mitochondrial function, which regulates sperm motility. An association between decreased sperm progressive motility and an increase in the percentage of spermatozoa with low MMP has been shown in both groups of diabetic patients compared to controls. Other studies have shown that mitochondrial ultrastructural alterations are associated with a decreased sperm motility (30, 31). In patients with DM1, the alteration of the mitochondrial function became evident in patients with a disease duration ranging between 5 and 10 years, whereas the alterations of sperm progressive motility became significant in patients with a disease duration greater than 10 years. Therefore, mitochondrial alteration seems to anticipate the subsequent and physiological decrease in sperm motility.

Sperm volume was lower in patients with DM2 although the difference did not reach the statistical significance, unlike to previously published data (28). This discrepancy may relate to the fact that patients with DM2 enrolled by Ali and colleagues (28) had lower TT values than the control group. In fact, the seminal vesicles that produce about 60% of the ejaculate volume are testosterone-dependent glands. In addition, patients with DM2 enrolled in the present study were younger, in childbearing age and not yet entirely affected by the age-related androgenic decline compared to those described in the literature. These DM2 patients enrolled had normal TT levels but higher values of LH compared to controls to indicate an initial testicular alteration. So, this slight reduction of the sperm volume may, however, represent the beginning of a process that could worsen at a later age. Instead, DM1 patients showed lower seminal fluid volume in the presence of normal blood circulating concentrations of androgens. The patients of our study have bigger epididymal head and tail diameters after ejaculation compared to controls, suggesting an alteration in the epididymal contractile function. This phenomenon, indicative of a dysfunctional epididymal voiding capability, may be the cause of the decreased seminal fluid volume observed in these patients.

The identification of leukocyte subpopulations in seminal fluid appears to acquire a greater clinical relevance to identify the cause of infection/inflammation (32). Leukocytes present in the rete testis, vas deferens, and human ejaculate are predominantly T-suppressor lymphocytes (33). The role of lymphocytes in the seminal fluid is not only antimicrobial but also to remove apoptotic or immature germ cells. The presence of these lymphocytes could improve seminal quality because T-suppressor lymphocyte removal action would avoid the activation of T-helper lymphocytes against antigen-presenting cells decreasing the production of antisperm antibodies and their possible sperm damage (32). Therefore, this could explain the increased percentage of T-suppressor lymphocytes in DM2 patients, where there is a greater number of apoptotic cells, while that of T-helper is decreased because of the absence of microbial agent and antigen-presenting cells.

With regards to oxidative stress indices, the mitochondrial superoxide anion concentration was found to be higher in DM2 than in DM1 patients. Indeed, LP increases only in DM2 patients, especially in relation to the higher concentration of seminal fluid leukocytes of this group of patients. In DM2 patients, the increase of LP and mitochondrial superoxide anion may decrease MMP and consequently be responsible for a greater sperm motility decline than in DM1.

Recently, it has been shown that the epididymis is a potential target of the oxidative stress in diabetic patients. Immunohistochemical analysis has shown the presence of AGE receptors in this organ (16). In addition, malonildialdehyde levels, the final product of LP, are higher in DM2 patients compared to controls (5, 6). These data are relevant to understand the physiopathological and clinical aspects that can address a more targeted and specific therapeutic approach. In addition, a greater percentage of DM2 patients are less vital, in late apoptosis and with fragmentation of sperm DNA. These data, associated with an increased number of leukocytes in the seminal fluid, with an increased LP and levels of mitochondrial superoxide, indicate that oxidative stress is the leading cause of impaired sperm quality of DM2 patients.

Finally, the didymo-epididymal ultrasound evaluation revealed a lack of physiological contraction of the cranial and caudal portion of the epididymis after ejaculation in DM1 patients. Conventionally, the ultrasound epididymal thickness decreases by approximately 3 mm after ejaculation, as found in DM1 patients with a duration of illness of less than 10 years. This decrease did not occur in patients with a long (>10 years) disease duration suggesting an atonia/hypotonia state of these anatomical structures, poorly studied in patients with DM1. Epididymal cranial and caudal tracts are characterized by a spontaneous peristalsis that can also carry immotile spermatozoa (34). The caudal portion of the epididymis has a rich adrenergic innervation, which is activated during ejaculation. Adrenergic denervation does not eliminate epididymal contractility (35). This suggests that the contractility is mediated by a neuronal mechanism and other hormonal (oxytocin and endothelin 1) and non-hormonal factors, which can alter the contractile function of the epididymis.

### Conclusive Remarks

Type 1 diabetes mellitus patients have low ejaculate volume due to a lack of physiological epididymal contraction associated with mitochondrial damage that anticipates the subsequent decline of sperm progressive motility. These findings are associated with an increased oxidative stress that can also alter other conventional sperm parameters, although to a lower extent than in DM2 patients.

Type 2 diabetes mellitus patients, instead, seem to be characterized by an amicrobial inflammatory condition and increased concentration of seminal fluid leukocytes, which raises oxidative stress indices damaging conventional sperm parameters, sperm DNA, and vitality. Since diabetic disease affects male fertility, the prevention of DM2 and the follow-up of seminal parameters in DM1 should be recommended in patients in childbearing age.

## Ethics Statement

This study was approved by the Ethics Committee of University teaching Hospital of “Policlinico-Vittorio Emanuele,” University of Catania (Catania, Italy). All methods were performed in accordance with the relevant guidelines and regulations. All participants were asked for and provided their informed consent.

## Author Contributions

RC is the principal investigator of this study. AC is the coordinator of all phases of this study. Other authors (SV, LM, and AA) were involved in methodological and statistical aspects.

## Conflict of Interest Statement

The authors declare that the research was conducted in the absence of any commercial or financial relationships that could be construed as a potential conflict of interest.

## References

[1] Hamilton BE Ventura SJ Fertility and abortion rates in the United States, 1960–2002. Int J Androl (2002) 29:34–45. 10.1111/j.1365-2605.2005.00638.x 16466522

[2] Lutz W. Fertility rates and future population trends: will Europe’s birth rate recover or continue to decline? Int J Androl (2006) 29:25–33. 10.1111/j.1365-2605.2005.00639.x 16466521

[3] Silink M. Childhood diabetes: a global perspective. Horm Res (2002) 57:1–5. 10.1159/000053304 11979014

[4] Lascar N Brown J Pattison H Barnett AH Bailey CJ Bellary S. Type 2 diabetes in adolescents and young adults. Lancet Diabetes Endocrinol (2018) 6:69–80. 10.1016/S2213-8587(17)30186-9 28847479

[5] La Vignera S Condorelli R Vicari E D’Agata R Calogero AE. Diabetes mellitus and sperm parameters. J Androl (2012) 33:145–53. 10.2164/jandrol.111.013193 21474785

[6] La Vignera S Condorelli RA Vicari E D’Agata R Salemi M Calogero AE. High levels of lipid peroxidation in semen of diabetic patients. Andrologia (2012) 44(Suppl 1):565–70. 10.1111/j.1439-0272.2011.01228.x 21919944

[7] Mallidis C Agbaje I O’Neill J McClure N. The influence of type 1 diabetes mellitus on spermatogenic gene expression. Fertil Steril (2009) 92:2085–7. 10.1016/j.fertnstert.2009.06.006 19589518

[8] Agbaje IM Rogers DA McVicar CM McClure N Atkinson AB Mallidis C Insulin dependant diabetes mellitus: implications for male reproductive function. Hum Reprod (2007) 22:1871–7. 10.1093/humrep/dem077 17478459

[9] Roessner C Paasch U Kratzsch J Glander HJ Grunewald S. Sperm apoptosis signalling in diabetic men. Reprod Biomed Online (2012) 25:292–9. 10.1016/j.rbmo.2012.06.004 22796231

[10] Lestienne P Reynier P Chretien MF Penisson-Besnier I Malthièry Y Rohmer V. Oligoasthenospermia associated with multiple mitochondrial DNA rearrangements. Mol Hum Reprod (1997) 3:811–4. 10.1093/molehr/3.9.811 9358008

[11] Silvestroni L Modesti A Sartori C. Insulin-sperm interaction: effects on plasma membrane and binding to acrosome. Arch Androl (1992) 28:201–11. 10.3109/01485019208987699 1530369

[12] Cameron DF Murray FT Drylie DD. Interstitial compartment pathology and spermatogenic disruption in testes from impotent diabetic men. Anat Rec (1985) 213:53–62. 10.1002/ar.1092130108 4073561

[13] Chan JL Mantzoros CS. Leptin and the hypothalamic-pituitary regulation of the gonadotropin-gonadal axis. Pituitary (2001) 4:87–92. 10.1023/A:1012947113197 11824513

[14] Pitteloud N Hardin M Dwyer AA Valassi E Yialamas M Elahi D Increasing insulin resistance is associated with a decrease in Leydig cell testosterone secretion in men. J Clin Endocrinol Metab (2005) 90:2636–41. 10.1210/jc.2004-2190 15713702

[15] Amaral S Oliveira PJ Ramalho-Santos J. Diabetes and the impairment of reproductive function: possiblerole of mitochondria and reactive oxygen species. Curr Diabetes Rev (2008) 4:46–54. 10.2174/157339908783502398 18220695

[16] Mallidis C Agbaje IM Rogers DA Glenn JV Pringle R Atkinson AB Advanced glycation end products accumulate in the reproductive tract of men with diabetes. Int J Androl (2008) 32:295–305. 10.1111/j.1365-2605.2007.00849.x 18217985

[17] Condorelli RA Calogero AE Vicari E Duca Y Favilla V Morgia G Prevalence of male accessory gland inflammations/infections in patients with type 2 diabetes mellitus. J Endocrinol Invest (2013) 36:770–4. 10.3275/8950 23633651

[18] Patterson JE Andriole VT. Bacterial urinary tract infections in diabetes. Infect Dis Clin North Am (1997) 11:735–50. 10.1016/S0891-5520(05)70383-4 9378933

[19] Guest CB Park MJ Johnson DR Freund GG. The implication of proinflammatory cytokines in type 2 diabetes. Front Biosci (2008) 13:5187–94. 10.2741/3074 18508580

[20] La Vignera S Di Mauro M Condorelli R La Rosa S Vicari E Diabetes worsens spermatic oxidative “stress” associated with the inflammation of male accessory sex glands. Clin Ter (2009) 160(5):363–6. 19997681

[21] La Vignera S Calogero AE Condorelli R Lanzafame F Giammusso B Vicari E. Andrological characterization of the patient with diabetes mellitus. Minerva Endocrinol (2009) 34(1):1–9. 19209124

[22] American Diabetes Association. Standards of medical care in diabetes – 2012. Diabetes Care (2012) 35:S11–63. 10.2337/dc12-s011 22187469 PMC3632172

[23] Zegers-Hochschild F Adamson GD Dyer S Racowsky C de Mouzon J Sokol R The international glossary on infertility and fertility care. Hum Reprod (2017) 32:1786–801. 10.1093/humrep/dex234 29117321 PMC5850297

[24] World Health Organization. WHO Laboratory Manual for the Examination and Processing of Human Semen. 5th ed Cambridge, UK: Cambridge University Press (2010). 2010 p.

[25] La Vignera S Condorelli RA Vicari E Salmeri M Morgia G Favilla V Microbiological investigation in male infertility: a practical overview. J Med Microbiol (2014) 63:1–14. 10.1099/jmm.0.062968-0 24072761

[26] Barbonetti A Vassallo MR Di Rosa A Leombruni Y Felzani G Gandini L Involvement of mitochondrial dysfunction in the adverse effect exerted by seminal plasma from men with spinal cord injury on sperm motility. Andrology (2013) 1:456–63. 10.1111/j.2047-2927.2013.00077.x 23494980

[27] Niven MJ Hitman GA Badenoch DF. A study of spermatozoal motility in type 1 diabetes mellitus. Diabet Med (1995) 12:921–4. 10.1111/j.1464-5491.1995.tb00397.x 8846685

[28] Ali ST Shaikh RN Siddiqi NA Siddiqi PQ Semen analysis in insulin-dependent/non-insulin-dependent diabetic men with-without neuropathy. Arch Androl (1993) 30:47–54. 10.3109/01485019308987744 8420505

[29] Padrón RS Dambay A Suarez R Màs J. Semen analyses in adolescent diabetic patients. Acta Diabetol Lat (1984) 21:115–21. 10.1007/BF02591100 6475450

[30] Amaral A Lourenço B Marques M Ramalho-Santos J Mithocondria functionality and sperm quality. Reproduction (2013) 146:163–74. 10.1530/REP-13-0178 23901129

[31] Pelliccione F Micillo A Cordeschi G D’Angeli A Necozione S Gandini L Altered ultrastructure of mithocondrial membranes is strongly associated with unexplained asthenozoospermia. Fertil Steril (2011) 95:641–6. 10.1016/j.fertnstert.2010.07.1086 20840880

[32] Seshadri S Flanagan B Vince G Lewis-Jones DJ. Detection of subpopulations of leucocytes in different subgroups of semen sample qualities. Andrologia (2012) 44:354–61. 10.1111/j.1439-0272.2011.01189.x 21806656

[33] Witkin SS. Mechanisms of active suppression of the immune response to spermatozoa. Am J Reprod Immunol Microbiol (1988) 17:61–4. 10.1111/j.1600-0897.1988.tb00204.x 2973252

[34] El-Badawi A Schenk EA The distribution of cholinergic and adrenergic nerves in the mammalian epididymis: a comparative histochemical study. Am J Anat (1967) 121:1–14. 10.1002/aja.1001210102 6069971

[35] Kempinas WD Suarez JD Roberts NL Strader L Ferrell J Goldman JM Rat epididymal sperm quantity, quality, and transit time after guanethidine-induced sympathectomy. Biol Reprod (1998) 59:890–6. 10.1095/biolreprod59.4.890 9746740

